# Inhibition of Vasculogenic Mimicry and Angiogenesis by an Anti-EGFR IgG1-Human Endostatin-P125A Fusion Protein Reduces Triple Negative Breast Cancer Metastases

**DOI:** 10.3390/cells10112904

**Published:** 2021-10-27

**Authors:** Seung-Uon Shin, Hyun-Mi Cho, Rathin Das, Hava Gil-Henn, Sundaram Ramakrishnan, Ahmed Al Bayati, Stephen F. Carroll, Yu Zhang, Ankita P. Sankar, Christian Elledge, Augustin Pimentel, Marzenna Blonska, Joseph D. Rosenblatt

**Affiliations:** 1 Sylvester Comprehensive Cancer Center, Department of Medicine, Division of Hematology, University of Miami Miller School of Medicine (UMMSOM), Miami, FL 33136, USA; sshin@med.miami.edu (S.-U.S.); hcho@med.miami.edu (H.-M.C.); ahmed.albayati@uky.edu (A.A.B.); y.zhang4@med.miami.edu (Y.Z.); MBlonska@med.miami.edu (M.B.); 2 Synergys Biotherapeutics Inc., Alamo, CA 94507, USA; rathindas@synergysbio.com (R.D.); sfcarroll@earthlink.net (S.F.C.); 3 The Azrieli Faculty of Medicine, Bar-Ilan University, Safed 1311502, Israel; Hava.Henn@biu.ac.il; 4 Department of Surgery, University of Miami Miller School of Medicine (UMMSOM), Miami, FL 33136, USA; sramakrishnan@miami.edu; 5 Kentucky Clinic, University of Kentucky, Lexington, KY 40536, USA; 6 Sheila and David Fuente Graduate Program in Cancer Biology, Sylvester Comprehensive Cancer Center, University of Miami Miller School of Medicine (UMMSOM), Miami, FL 33136, USA; aps195@miami.edu (A.P.S.); Mce58@med.miami.edu (C.E.); 7 Sylvester Comprehensive Cancer Center, Department of Medicine, Division of Medical Oncology, University of Miami Miller School of Medicine (UMMSOM), Miami, FL 33136, USA; apimentel@med.miami.edu

**Keywords:** EGFR, endostatin, vasculogenic mimicry, triple negative breast cancer

## Abstract

Triple negative breast cancer (TNBC) is an aggressive breast cancer subtype with limited therapeutic options. Metastasis is the major cause of TNBC mortality. Angiogenesis facilitates TNBC metastases. Many TNBCs also form vascular channels lined by tumor cells rather than endothelial cells, known as ‘vasculogenic mimicry’ (VM). VM has been linked to metastatic TNBC behavior and resistance to anti-angiogenic agents. Epidermal growth factor receptor (EGFR) is frequently expressed on TNBC, but anti-EGFR antibodies have limited efficacy. We synthesized an anti-EGFR antibody–endostatin fusion protein, αEGFR IgG1-huEndo-P125A (αEGFR-E-P125A), designed to deliver a mutant endostatin, huEndo-P125A (E-P125A), to EGFR expressing tumors, and tested its effects on angiogenesis, TNBC VM, and motility in vitro, and on the growth and metastasis of two independent human TNBC xenograft models in vivo. αEGFR-E-P125A completely inhibited the ability of human umbilical vein endothelial cells to form capillary-like structures (CLS) and of TNBC cells to engage in VM and form tubes in vitro. αEGFR-E-P125A treatment reduced endothelial and TNBC motility in vitro more effectively than E-P125A or cetuximab, delivered alone or in combination. Treatment of TNBC with αEGFR-E-P125A was associated with a reduction in cytoplasmic and nuclear β-catenin and reduced phosphorylation of vimentin. αEGFR-E-P125A treatment of TNBC xenografts in vivo inhibited angiogenesis and VM, reduced primary tumor growth and lung metastasis of orthotopically implanted MDA-MB-468 TNBC cells, and markedly decreased lung metastases following intravenous injection of MDA-MB-231-4175 lung-tropic TNBC cells. Combined inhibition of angiogenesis, VM, and TNBC motility mediated by αEGFR-E-P125A is a promising strategy for the prevention of TNBC metastases.

## 1. Introduction

Angiogenesis, the development of new blood vessels by endothelial cells, is essential for neoplastic progression. Tumor vasculature may also be assembled through the formation of vascular channels lined by clonally related malignant tumor cells, a process known as ‘vasculogenic mimicry’ (VM) [1,2,3,4,5]. Many cancers exhibit VM, and evidence for VM includes formation by tumor cells of capillary-like structures in vitro and histologic demonstration of tumor cell-lined vessels in human cancers, including ocular and cutaneous melanoma, ovarian cancer [1,2,3,4], and TNBC [5,6]. VM has been linked to metastatic potential and may contribute to resistance to anti-angiogenic agents, and many angiogenesis inhibitors have little/no effect on VM [3,4,5,7,8]. VM may also contribute to elevated tumor oncotic pressure that impedes chemotherapeutic drug delivery [7,9].

TNBC is a highly lethal form of breast cancer. TNBC accounts for 15–20% of breast cancer cases and is associated with early relapse and poor prognosis [10,11]. VM is frequently seen in TNBC and may play an important role in TNBC pathogenesis and resistance to anti-angiogenic agents [7,9]. VEGF blockade with sunitinib in TNBC xenografts promotes increased VM, and ‘rebound angiogenesis’ is seen following sunitinib withdrawal [7]. We hypothesized that combined inhibition of both VM and angiogenesis would increase the efficacy of anti-TNBC therapy.

Endostatin, a 22 kD cleavage product of collagen XVIII, inhibits multiple angiogenic pathways and tumor growth in murine tumor and xenograft models [12,13,14]. Although endostatin inhibits angiogenesis, it does not inhibit VM [5,6,8]. We previously linked wild type murine, human endostatin, or mutant human endostatin with increased anti-angiogenic properties containing a proline to alanine substitution at amino acid 125 (E-P125A) to a humanized anti-HER2 IgG3 antibody (αHER2-E-P125A) [15,16,17]. αHER2-E-P125A demonstrated longer t_1/2_ than endostatin in vivo, a superior anti-angiogenic efficacy, and greater anti-tumor efficacy than anti-HER2 antibody, endostatin, or αHER2-fused to wild type endostatin against HER2+ breast cancer xenografts [15,16]. We have now synthesized and tested a second antibody fusion protein directed against the human EGFR receptor, which is commonly expressed in a wide variety of human epithelial malignancies.

Approximately 80% of TNBC may demonstrate basal-like gene expression. Many basal-like breast cancers express EGFR [11]. Basal-like breast cancers frequently demonstrate evidence of epithelial-mesenchymal transition and have a high frequency of EGFR amplification [18]. Despite frequent expression of EGFR in TNBC, trials of the anti-EGFR antibody cetuximab in metastatic TNBC demonstrated little/no efficacy [19,20].

In this report, we studied the ability of an anti-EGFR IgG1 antibody fused to a mutant form (E-P125A) of human endostatin, αEGFR-E-P125A, to inhibit angiogenesis, TNBC VM, motility, and TNBC xenograft growth/metastasis in vivo and the investigated underlying mechanisms.

## 2. Materials and Methods

### 2.1. Cell Lines, Materials, and Animals

MDA-MB-231 and MDA-MB-468 TNBC cells were purchased from ATCC (Manassas, VA, USA). The lung tropic MDA-MB-231-4175 was a gift from J. Massagué, MSKCC [21]. TNBC lines were cultured in RPMI-1640/10% FBS and 100 U/mL penicillin–streptomycin (Gibco-BRL).

Early [3,4,7] passage human umbilical vein endothelial cells (HUVEC) (ATCC, Manassas, VA, USA), cultured in EGM-2 (Lonza; Walkersville, MD, USA), were used in all experiments. Other materials are listed in the Supplementary Table S1.

### 2.2. Construction and Expression of αEGFR IgG1-huEndo-P125A (αEGFR-E-P125A) and Fc-huEndo-P125A (Fc-E-P125A)

By using the publicly available amino acid sequences of Cetuximab (http://www.drugbank.ca/drugs/DB00002, accessed on 11 September 2007), coding sequences for the heavy and light chains were deduced using the Vector NTI program and synthesized, and heavy and light chain variable region genes were subcloned into human IgG3 and human IgG3-huEndo expression vectors as previously described [16]. An αEGFR IgG1-huEndostatin-P125A heavy chain gene was constructed by using αEGFR IgG3-huEndostatin-P125A heavy chain coding sequences as a template and replacing the IgG3 hinge region with an GAGCCCAAATCTTGTGACAAAACTCACACATGCCCACCGTGCCCA sequence encoding the IgG1 hinge region by PCR. Anti-EGFR IgG1-huEndostatin-P125A heavy chain and kappa light chain gene coding sequences were cloned separately into SMART Expression pCT™ vectors (Celltheon) and co-transfected into CHO cells. Stably transduced CHO cells expressing αEGFR IgG1-huEndo-P125A (αEGFR-E-P125A) were selected, and fusion protein was purified from culture supernatants using a Mab Select SuRe column (GE Healthcare Life Sciences) followed by SE-HPLC, which resulted in >95% purity as determined by Western blots using the anti-IgG and anti-endostatin sera and SEC chromatography (fusion yield: ~400 mg/L).

To generate Fc-E-P125A, which lacked EGFR targeting sequences, αEGFR-E-P125A heavy chain coding sequences were used as a template and Fab coding sequences were removed by PCR. The remaining Fc-E-P125A sequence was subcloned into the SMART Expression pCT™ vectors (Celltheon), transfected into CHO cells, and purified from the supernatant as stated above.

Human recombinant endostatin was purchased from Genscript (Piscataway, NJ, USA). Human endostatin P125A (E-P125A) was expressed in *Pichia pastoris* and purified using heparin linked ceramic particles as previously described [16].

### 2.3. Endothelial Capillary-Like Tube Formation Formation and Vasculogenic Mimicry Formation Assays in Matrigel

Tube formation (CLS) using human endothelial cells and vasculogenic mimicry (VM) assays using TNBC tumor cells were carried out per previously published methods [16]. HUVEC or MDA-MB-231, MDA-MB-231-4175, or MDA-MB-468 cells were resuspended in EGM2 or RPMI (Lonza, Walkersville, MD) and plated on matrigel-coated wells containing αEGFR-E-P125A or control proteins as indicated. Following 8–16 h at 37 °C, tube formation was examined and photomicrographs of the center of each well obtained at 50 fold magnification. CLS formation by HUVEC or MDA-MB-231-4175 was recorded at 30-min intervals through an inverted photomicroscope for 15 h using Cellomics time-lapse photography. Using NIH ImageJ software, the tracking of migration by ten randomly selected cells over 15 h was plotted, and the cumulative distance traversed by selected cells was calculated.

### 2.4. Cell Migration Assay

HUVECs or MDA-MB-231 cells were incubated for 8 h in serum-free media containing αEGFR-E-P125A or equimolar control proteins in the top chamber of a transwell plate (CIM-16; ACEA Biosciences, Inc., San Diego, CA, USA). The bottom chamber contained 10% FBS/EGM-2 as a chemoattractant. Impedance from a plate sensor was measured using the xCELLigence RTCA-SP system (ACEA Biosciences, Santa Clara, CA, USA) every 15 min.

### 2.5. Scratch Wound Migration Assay

MDA-MB-231-4175 or HUVEC were plated in the 96-well ImageLock™ Microplate (Essen BioScience, Ann Arbor, MI, USA) at a density of 3.5 × 10^4^ cells per well in RPMI or EGM2 medium, respectively. Then, 24 h after seeding, WoundMaker™ (Essen BioScience Ann Arbor, Mich., USA) was used to create reproducible scratch wounds in each well. Media were aspirated after wounding, and cells were washed with fresh media and treated with αEGFR-E-P125A or equimolar amounts of control treatments. The assay plate was incubated in an Incucyte ZOOM^®^ (Essen BioScience), and wound width was monitored by imaging at the 10x objective every 4 h. Wound width was characterized by the average distance between the top and bottom edges of the migrating cell population within the scratch wound (microns) and was computed by the Incucyte software.

### 2.6. Mosaic Vessel Formation by Endothelial (HUVEC) and TNBC (MDA-MB-231-4175, or MDA-MB-468) Cells Plated in Combination

HUVECs were labeled with CellTrace™ Calcein Red-Orange AM in EBM2 medium (Lonza, Morrisville, NC, USA) supplemented with 2% FBS and MDA-MB-231-4175 with CellTrace™ calcein green AM (Thermo Fisher Scientific, Waltham, MA, USA) in RPMI supplemented with 10% FBS per the manufacturer’s protocol. HUVEC and MDA-MB-231-4175 cells (5 × 10^4^) were re-suspended with serum-free EBM-2 for endothelial cells and RPMI for TNBC cells. For mosaic vessel formation assay, tumor cells and HUVEC were co-cultured together in serum-free EBM2 and RPMI on matrigel for 16 h and treated with αEGFR IgG1-huEndo-P125A or controls as indicated. Each condition group contained three wells.

In a second experiment, HUVEC were labeled with CellTracker™ Red CMTPX and MDA-MB-468 cells with CellTracker™ Green CMFDA, cultured alone, or for ‘mosaic’ vessel formation assays, tumor cells and HUVEC were co-cultured together on matrigel for 16 h, treated as indicated, and imaging was performed with a Leica confocal fluorescent microscope.

### 2.7. TCF/LEF-1 Luciferase Reporter Gene Assay

MDA-MB-231 cells were seeded into 96-well plates pre-coated with matrigel at 10^4^ cells/well. Then, 24 h after seeding, Cignal lentiviral particles (Cignal Lenti TCF/LEF Reporter, luc) encoding the TCF/LEF-1 reporter gene (Qiagen, Germantown, MD, USA) were added and incubated for 18–20 h at 37 °C, and puromycin (2 µg/mL) was added to select stable reporter containing cells.

MDA-MB-231 (TCF/LEF Luc Reporter) cells (1 × 10^4^ cells/well) were plated in 96-well plates pre-coated with matrigel or left uncoated and treated with equimolar E-P125A (58 μg/mL), cetuximab (170 μg/mL), or αEGFR-E-P125A (250 μg/mL) for 16 h at 37 °C. Luciferase activity was measured using Luciferin and a luminometer (ThermoFisher Labsystems, Frederick, MD, USA). Relative luciferase activity (RLA, %) was expressed as the ratio of treatment to media.

### 2.8. In Vivo Animal Tumor Experiments

MDA-MB-468 cells (5 × 10^6^/mouse) were injected into the mammary fat pad of female NSG mice (Jackson Laboratory, Bar Harbor, ME, USA). Starting day 5 post tumor implantation, mice were intravenously treated with PBS, cetuximab, or αEGFR-E-P125A fusion protein at the indicated intervals (*n* = 8 mice per treatment group). Tumor size was assessed by a digital caliper.

To evaluate αEGFR-E-P125A efficacy on pulmonary metastasis, 5 × 10^4^ lung tropic MDA-MB-231-4175 cells expressing luciferase were injected into the tail vein of NSG mice. Mice were treated by intravenous injection starting on day 5 with αEGFR-E-P125A, cetuximab, or PBS 2x weekly. Tumor growth/metastases were monitored using an In Vivo Imaging System (IVIS, PerkinElmer).

Mice were euthanized for distress, abnormal behavior, and at experiment termination. Following euthanasia, primary tumors and/or lungs were collected and mounted in OCT (Sakura Finetek, Torrance, CA, USA) for immunohistochemical staining and were frozen as outlined below.

All animal experiments were conducted in compliance with the NIH Guidelines for the Care and Use of Laboratory Animals as approved by the University of Miami Institutional Animal Care and Use Committee.

### 2.9. Immunohistochemical Staining and Quantification

Primary tumor sections and/or lungs were excised from euthanized mice and frozen in liquid nitrogen, and 8 μm frozen sections were prepared. Tumor sections were stained with diaminobenzidine (DAB) and hematoxylin, and the relative positive optical density/total optical density of staining was quantified using NIH ImageJ software [22]. Representative sections from a tumor selected for ‘average’ photon intensity are shown. For example, cryosections from representative treated mice were stained with anti-murine CD31 or anti-human Laminin. Representative lung sections were from treated PBS (mouse #3) (3.24 × 10^6^ p/s/cm^2^/sr, average: 6.60 × 10^6^ p/s/cm^2^/sr), cetuximab (mouse #1) (2.47 × 10^6^ p/s/cm^2^/sr, average: 2.65 × 10^6^ p/s/cm^2^/sr), and αEGFR-E-P125A (#4) (2.36 × 10^5^ p/s/cm^2^/sr, average: 3.20 × 10^5^ p/s/cm^2^/sr). An immunohistochemically stained image was opened under ‘Colour Deconvolution’ and ‘H DAB’ for the stain chosen. The ‘Colour 2′ image was quantified as DAB+ stain, and the original image was measured as a ‘total’ stain; intensity measurements were converted to ‘Optical Density (OD)’, and the relative positive stain (%) was calculated dividing by the total OD with the following formula: OD = log (Max. intensity/Mean intensity), where max. intensity = 255 for 8-bit images. Percent (%) = positive OD/total OD × 100 [22].

### 2.10. MMP2 and MT1-MMP ELISA

Secreted MMP-2 and soluble MT1-MMP protein from supernatants of culture samples in the presence or absence of matrigel were determined using ELISA kits (Abcam, San Francisco, CA, USA) per the manufacturer’s instructions.

### 2.11. Antibody Dependent Cellular Cytotoxicity (ADCC) Assays: ADCC Activity

ADCC activity was determined using a FACS based ADCC assay. Fresh PBMCs purified from normal blood donors served as effector cells. CFSE labeled target cells (MDA-MB-231, MDA-MB-468 and MDA-MB-231, and A431) and PBMC were used at a ratio of 1:50. Indicated antibodies or fusion proteins, and target-effector cell mixture were incubated in triplicate at 37 °C for 4 h. Non-specific IgG1 and Fc-E-P125A were used as negative controls. Cells from the wells were transferred to a FACS tubes containing 0.5% BSA in PBS (pH 7.4) and propidium iodide (PI, 2μg/mL), and cell killing analyzed by flow cytometry (iQue Screener, Intellicyt or BD FACScan, BD Biosciences). ADCC was assessed by measuring frequency of dead target cells (CFSE+/PI+)/ total target cells in the sample (CFSE+/PI+ and CFSE+/PI-). Co-ordinates noted in X-axis denote antibody or fusion concentration ranging from 0.04 to 30 μg/mL while % dead cells are noted in the Y-axis. Details are in Methods

### 2.12. Statistical Analysis

Statistical analysis was carried out with Graphpad Prism 7.03 (GraphPad Software, Inc., La Jolla, CA). Tube formation, migration, and the metastasis and the survival rates in human TNBC xenografts were analyzed using the two-way ANOVA test of GraphPad Prism 7.03. IHC comparison, western blotting, luciferase activity, and MMP assays were analyzed using the one-way ANOVA test of GraphPad Prism 7.03. Results are presented as the mean ± SD unless otherwise specified. Differences were considered statistically significant at *p* < 0.05.

## 3. Results

### 3.1. Construction of Anti-EGFR IgG1-Human Endostatin-P125A (αEGFR-E-P125A) and Fc-E-P125A Fusion Proteins

Our prior studies using an anti-HER2 IgG3 antibody–endostatin fusion protein showed that replacement of native endostatin in the fusion with a human endostatin containing a proline to alanine mutation at position 125 (E-P125A) demonstrated enhanced anti-angiogenic and anti-tumor activity in both murine (EMT6-HER2) and human HER2 expressing SK-BR-3 xenograft models compared to fusions incorporating native endostatin [16]. In an effort to target basal-like TNBC and other EGFR expressing solid tumors, we have now fused E-P125A to anti-EGFR targeting sequences.

**Figure 1:**
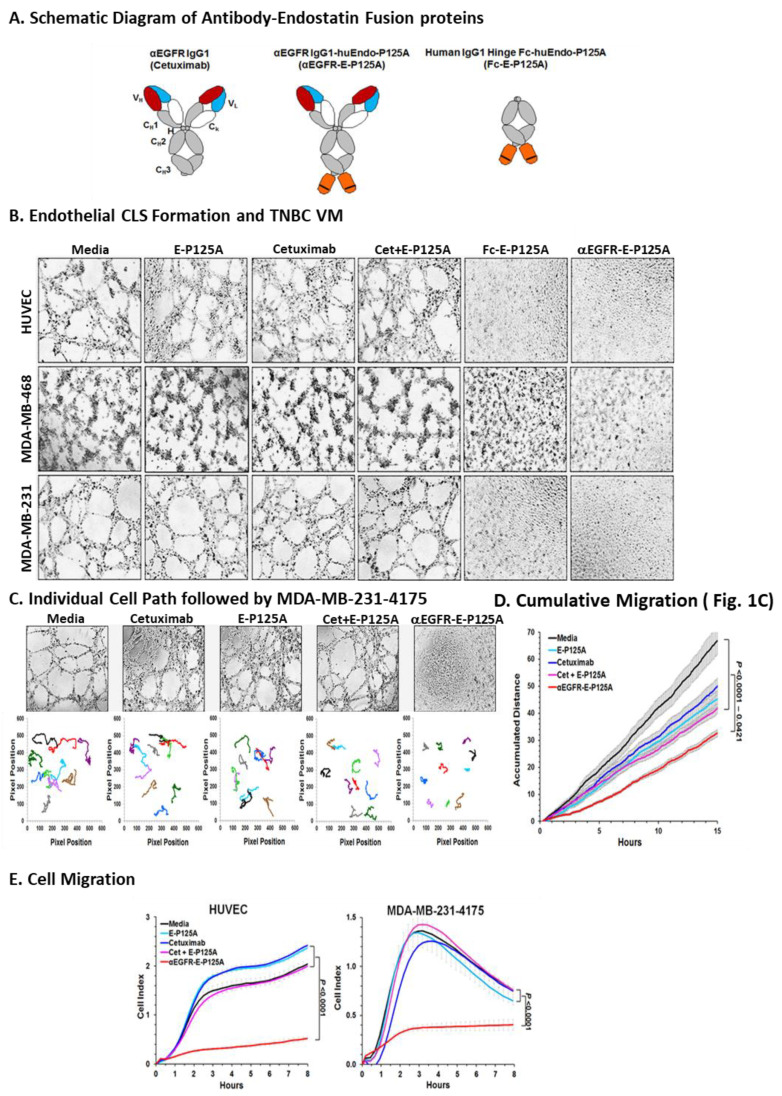
αEGFR-E-P125A: Effects on angiogenesis and VM. (**A**) Schematic diagram of antibody–endostatin fusion proteins (αEGFR-E-P125A and Fc-E-P125A): Human endostatin-P125A domains are shown in orange, and the proline to alanine mutation at amino acid 125 of endostatin is indicated by a black line. Details in methods. (**B**) Inhibition of HUVEC CLS formation and TNBC VM. HUVEC, MDA-MB-231, MDA-MB231-4175, or MDA-MB-468 cells were resuspended in EBM2 and RPMI, plated on matrigel, and treated as indicated for 16 h. Tube formation was recorded through an inverted photomicroscope. Conditions: Media, endostatin-P125A (E-P125A, 90.92 nmol/L), Cetuximab (Cet, 45.46 nmol/L), Combination of 90.92 nmol/L E-P125A and 45.46 nmol/L cetuximab (E-P125A + Cet), Fc-E-P125A (45.46 nmol/L), and αEGFR-E-P125A (45.46 nmol/L). Note that αEGFR-E-P125A and Fc-E-P125A each contain two fused E-P125As per molecule of the fusion protein, so 2x the concentration of E-P125A (90.92 nmol/L) was compared to indicated fusion proteins (at 45.46 nmol/L). C-D. Vasculogenic mimicry by MDA-MB-231-4175 and individual cell migration paths using time-lapse photography. (**C**) tube formation (VM) by MDA-MB-231-4175 under indicated conditions was observed at 30-min intervals for 15 h. Migration path of ten randomly selected cells from 0–15 h. (**D**) Cumulative traversed distance by 10 randomly selected TNBC cells from 0–15 h in Figure 1C. Data shown as the mean ± SEM. (**E**) Effects on HUVEC and MDA-MB-231-4175 migration: HUVEC (2 × 10^4^) and MDA-MB-231-4175 cells (5 × 10^4^) were incubated for 8 h in serum-free media containing an equimolar concentration (45 nM) of cetuximab, E-P125A, a combination of cetuximab and E-P125A, or αEGFR-E-P125A, in the top chamber of a transwell plate; the bottom chamber contained 10% FBS/EBM-2 as a chemoattractant. Migration-induced impedance changes were measured using xCELLigence RTCA-SP every 15 min. HUVEC and MDA-MB-231-4175 cells migrated to the bottom surface of the upper chamber within 3 h. HUVEC remained adherent for up to 8 h, while MDA-MB-231 cells gradually detached from the transwell bottom. Data are shown as the mean ± SEM.

Since antibody-dependent cellular cytotoxicity (ADCC) is known to contribute to the anti-tumor activity of cetuximab, we constructed an anti-EGFR fusion protein using an IgG1 backbone. Coding sequences for E-P125A were joined to the 3′ end of an anti-EGFR IgG1 heavy chain gene [16]. Resulting αEGFR-E-P125A heavy chain and an αEGFR kappa light chain expression vectors were stably transfected into CHO cells. The complete αEGFR-E-P125A fusion protein of ~220 kDa, an IgG1 antibody fused to two E-P125A molecules, was purified from the culture supernatant (Figure 1A).

As a control for the effects of EGFR targeting, a hinge-Fc-huEndo-P125A (Fc-E-P125A) fusion protein lacking the variable heavy chain targeting sequences of αEGFR IgG1 was also constructed and produced in a similar manner (Figure 1A and Supplementary Figure S1A). Expression of IgG1 and E-P125A domains and heavy and light chains of expected MW were verified by western blotting (Figure S1A).

Both cetuximab and αEGFR-E-P125A bound to EGFR-expressing MDA-MB-231, MDA-MB-231-4175, and MDA-MB-468 TNBC cells. αEGFR-E-P125A binding was greater than that seen with cetuximab, presumably due to additional binding through the dimeric E-P125A domain (Supplementary Figure S1B,C). αEGFR-E-P125A showed the highest level of binding to HUVEC (MFI 280.26). Cetuximab showed very low levels of binding (MFI 103.38 to human umbilical vein endothelial cells (HUVEC) by flow cytometry No binding of anti-human IgG was seen in the absence of a primary antibody (Supplementary Figure S1C). αEGFR-E-P125A and Fc-E-P125A binding to HUVEC or TNBC could also be detected using PE-conjugated anti-human endostatin antibody (Supplementary Figure S1D).

### 3.2. αEGFR-E-P125A Retains the Ability to Mediate ADCC

In addition to effects on EGFR signaling, the ability to mediate ADCC is an important contributor to cetuximab anti-tumor activity [23,24]. Since the fused endostatin domain might theoretically impair ADCC, we tested the ability of αEGFR-E-P125A to mediate ADCC using MDA-MB-468 and MDA-MB-231 as target cells.

Initially, binding studies were carried out using αEGFR-E-P125A and target cells by flow cytometry. Results showed significantly higher binding of αEGFR-E-P125A to MDA-MB-468 than to MDA-MB-231 (Supplementary Figure S2A). Binding of Fc-E-P125A was similar to the negative control since it lacked the anti-EGFR IgG1 sequence. Binding of cetuximab to EGFR+ A431 cells served as a positive control for EGFR binding (Supplementary Figure S2A).Significantly higher binding by cetuximab was seen to both A431 and MDA-MB-468, both higher expressers of EGFR, than to MDA-MB-231.

ADCC activity against MDA-MB-468 and MDA-MB-231 target cells was determined using a FACS-based ADCC assay. Normal donor peripheral blood mononuclear cells (PBMC) freshly purified from normal blood donors served as effector cells. CFSE-labeled target cells (MDA-MB-231 or MBA-MD-468) and PBMC effector cells were used at an effector to target ratio of 50:1. Non-specific IgG1 and Fc-E-P125A were used as negative controls. Potent ADCC activity was seen following 4 h of treatment with either cetuximab or αEGFR-E-P125A against MDA-MB-468 (high EGFR expression) and against MDA-MB-231 (lower EGFR expression) (Supplementary Figure S2B). No ADCC was seen with Fc-E-P125A lacking EGFR-targeting domains (Supplementary Figure S2B). Therefore, αEGFR-E-P125A retained the ability to mediate ADCC against EGFR-expressing targets.

### 3.3. αEGFR-E-P125A Inhibits Endothelial Angiogenesis and TNBC VM In Vitro

#### 3.3.1. In Vitro Angiogenesis

HUVEC assemble into capillary-like structures (CLS) when plated for 8–16 h on Matrigel in endothelial growth medium (EGM) (Figure 1B). Neither cetuximab nor E-P125A added alone, or an equimolar combination of cetuximab and E-P125A, significantly inhibited endothelial CLS formation. In contrast, when treated with αEGFR-E-P125A for 8–16 h at 37 °C, HUVEC failed to form CLS and remained completely dispersed. Treatment of HUVEC with Fc-E-P125A, which lacks EGFR-targeting sequences, also markedly inhibited CLS formation, suggesting that dimeric presentation of E-P125A was sufficient for the inhibition of HUVEC CLS formation.

#### 3.3.2. Vasculogenic Mimicry (VM)

While tumors are known to induce angiogenesis by surrounding normal endothelial cells, many tumors directly engage in the formation and lining of vascular channels, a process known as vasculogenic mimicry (VM) [1,2,3]. Both MDA-MB-468 and MDA-MB-231-4175 TNBC cells assembled into a network of tubular structures within 8–12 h of plating on Matrigel, a surrogate assay for VM. Both αEGFR-E-P125A and Fc-E-P125A treatment for 16 h completely eliminated VM in vitro (Figure 1B). VM was not inhibited by treatment with equimolar E-P125A, cetuximab, or by the combination of cetuximab and E-P125A (Figure 1B).

### 3.4. αEGFR-E-P125A Treatment of HUVEC or TNBC Inhibits Cell Migration

Using time-lapse photography, we studied the paths and distance traversed by 10 randomly selected MDA-MB-231-4175 cells during VM/tube formation (Figure 1C). αEGFR-E-P125A significantly reduced the cumulative distance migrated by ten randomly selected cells relative to media (*p* < 0.0001), cetuximab (*p* = 0.0005), E-P125A (*p* = 0.0098), or the combination of cetuximab + E-P125A (*p* = 0.0339) (Figure 1C,D).

We next directly measured the migration of both HUVEC and TNBC through a transwell membrane into a lower chamber containing EGM, in the presence of either αEGFR-E-P125A or control treatments using an xCELLigence RTCA-SP system (Figure 1F). αEGFR-E-P125A significantly reduced migration by HUVEC (*p* < 0.0001) or MDA-MB-231-4175 cells, compared to media, cetuximab, E-P125A, or the combination (*p* < 0.0001) (Figure 1E).

Migration of both HUVEC and TNBC in the presence of αEGFR-E-P125A was additionally characterized by measuring wound closure in a scratch wound assay. HUVEC or MDA-MB-231-4175 TNBC was wounded, treated with either αEGFR-E-P125A or controls, and periodically imaged for wound width using the Incucyte Zoom^®^ system (Essen BioScience, Ann Arbor, Mich., USA) for 16 h. αEGFR-E-P125A treatment resulted in the near complete inhibition of wound closure and a significantly larger endpoint wound width compared to the controls in both HUVEC and TNBC (Supplementary Figure S3).

Therefore, the formation of CLS by HUVEC and/or formation of tube-like structures by TNBC (VM) were completely inhibited in vitro by αEGFR-E-P125A, and inhibition was associated with a marked reduction in motility of both HUVEC and TNBC cells.

### 3.5. αEGFR-E-P125A Inhibits ‘Mosaic ’ Tube Formation in Combined HUVEC-TNBC Co-cultures

In addition to VM, ‘mosaic’ vascular channels incorporating both tumor and endothelial cells have been described in vivo in breast and other cancers [2,25,26,27]. Silvestri et al. estimated that approximately 6% of breast cancer vessels may incorporate both tumor cells and endothelial cells in mouse xenograft models as well as in human tumors [26]. While the overall role of mosaic vessels in tumor progression is unknown, assembly of such ‘mosaic vasculature’ is thought to play an important role in development of a vascular supply.

**Figure 2:**
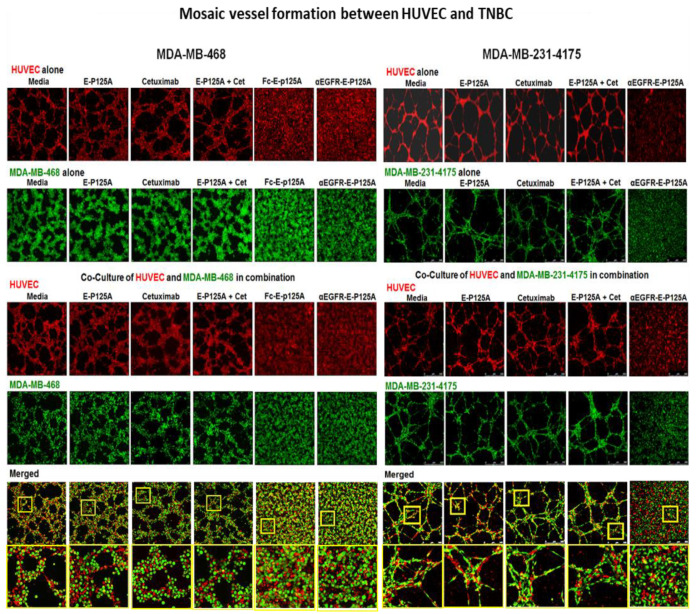
αEGFR-E-P125A inhibition of ‘mosaic’ vessel formation: HUVEC and TNBC co-culture. Left Panel HUVECs labeled with CellTracker™ Red CMTPX and MDA-MB-468 with CellTracker™ Green CMFDA. HUVECs (left panel, top row) in serum-free EBM2 or MDA-MB-468 (left panel, second row) in serum-free RPMI cultured alone on matrigel for 16 as indicated: αEGFR-E-P125A, or controls (media, E-P125A, cetuximab, or the combination of E-P125A and cetuximab (E-P125A + Cet)). MDA-MB-468 and HUVEC in serum-free EBM2 and RPMI co-cultured on matrigel for 16 h with indicated treatment (Left panel bottom four rows). Fluorescent imaging is shown for HUVEC (red), MDA-MB-468 (green), or both (merged). Right Panel: HUVEC and MDA-MB-231-4175 co-culture (HUVEC labeled with CellTrace™ Calcein Red-Orange AM (red) or MDA-MB-231-4175 labeled with CellTrace™ calcein green AM (green) were cultured separately on matrigel for 16 h and treated with αEGFR-E-P125A or controls [media, E-P125A, cetuximab, or E-P125A and cetuximab combined (E-P125A + Cet)]. (Right panel, top two rows). Labeled MDA-MB-231-4175 and HUVEC cocultured on matrigel (Rightpanel, bottom 4 rows). Labelled MDA-MB-231-4175 and HUVEC co-cultured on matrigel 16 h and treated as indicated Images taken with a Leica fluorescence confocal microscope. (Left and Right bottom panels): Higher magnification views are provided of areas highlighted in yellow bordered squares below the coculture figures in the, demonstrating the alignment and coalescence of HUVEC (red) and TNBC cells (green) in mosaic vessels, as well as the more random distribution of HUVEC and TNBC in cocultures exposed to αEGFR-E-P125A.

To test whether HUVEC and TNBC form mosaic vascular channels, we plated either MDA-MB-468 or MDA-MB-231-4175 TNBC cells alone or together with HUVEC (Figure 2). HUVEC were viably labeled with CellTrace™ Calcein Red-Orange AM (red), and MDA-MB-231-4175 or MDA-MB-468 TNBC cells were labelled with CellTrace™ calcein green AM (green). As seen previously (Figure 1), formation of CLS by HUVEC or assembly of VM tubular structures by MDA-MB-231-4175 or MDA-MB-468 plated independently was completely inhibited by αEGFR-E-P125A (Figure 2). When TNBC cells and HUVEC were plated together, HUVEC plated with either MDA-MB-468 or with MDA-MB-231-4175 TNBC formed overlapping ‘mosaic’ vascular structures on matrigel. The formation of such ‘mosaic’ CLS was completely inhibited by αEGFR-E-P125A (Figure 2). Higher magnification views of cocultures of HUVEC and either MDA-MB-468, or MDA-MB231-4175 TNBC demonstrated the non-random alignment and coalescence of HUVEC and TNBC into a lattice-like network of mosaic tubes. However, treatment with either Fc-E-P125A or αEGFR-E-P125A resulted in the loss of the well-structured mosaic tubes and a near random distribution of both cell types, HUVEC and TNBC (Figure 2). Therefore αEGFR-E-P125A completely inhibited ‘mosaic’ tube formation in combined HUVEC-TNBC co-cultures.

### 3.6. αEGFR-E-P125A Reduced Serine 39 Phosphorylation of Vimentin

TNBC are known to demonstrate a propensity to metastasize and frequently exhibit markers of epithelial to mesenchymal transition (EMT) [28,29]. Expression of EMT-associated markers including vimentin has been linked to poor prognosis in triple negative breast cancer [30]. Vimentin is a cytoskeletal protein that is involved in the migration of epithelial cells, and vimentin expression has been linked to EMT, and vimentin expression is increased in triple negative breast cancers [28,29]. VM involves TNBC transition from epithelial to endothelial behavior (EET). Many of the changes associated with EET and VM appear to be closely related to epithelial to mesenchymal transition or EMT [5]. Epithelial to endothelial transition (EET) shares many phenotypic characteristics of EMT, and hypoxia can promote VM in renal cell and non-small cell carcinoma [31] and is associated with TNBC EET, EMT, and VM [6,32,33,34]. MDA-MB-231-4175 cells and MDA-MB-231 cells demonstrate extensive epithelial to mesenchymal transition (EMT), which has been associated with TNBC invasive behavior, metastatic spread, and VM. Levels of vimentin in MDA-MB-231-4175 cells and MDA-MB231 were considerably higher as determined by Western blot and immunohistochemistry in MDA-MB-231-4175 cells and MDA-MB-231 than in MDA-MB-468, which retained epithelial characteristics and did not undergo similar mesenchymal differentiation.

Vimentin may also play a role in regulating angiogenesis by specifically binding to pro-angiogenic ligands such as Jagged, the ligand for NOTCH1 [35]. Vimentin activity is regulated by AKT, which phosphorylates vimentin on serine residue 39 (SER39), enhances vimentin effects on motility, and inhibits caspase induction of vimentin proteolysis [36]. SER39 phosphorylation also promotes vimentin disassembly and the release of fragments that facilitate the translocation of matrix metalloproteinases such as MT1-MMP, leading to ECM degradation and tissue invasion [36,37]. Vimentin cleavage and phosphorylation have also been associated with endothelial ‘sprout’ formation, a key step in the development of branching vasculature [37,38].

**Figure 3:**
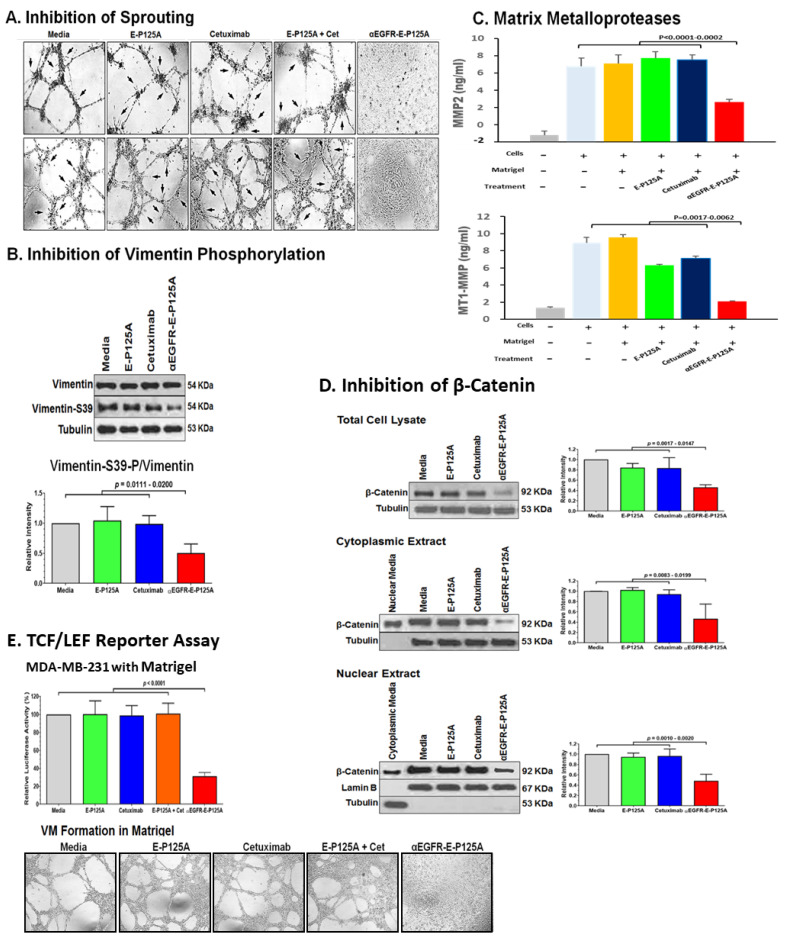
(**A**) Effects of αEGFR-E-P125A on sprouting: Tube formation by HUVECs or MDA-MB-231-4175 plated on matrigel, untreated or treated with E-P125A (58 μg/mL), cetuximab (170 μg/mL, Cet), a combination of E-P125A (58 μg/mL) and cetuximab (170 μg/mL), or αEGFR-E-P125A (250 μg/mL) for 16 h. Sprouting indicated by black arrows. (**B**) Western blot analysis for vimentin and SER39-phosphorylated vimentin following αEGFR-E-P125A treatment: MDA-MB-231-4175 cells plated in matrigel were treated with E-P125A (58 μg/mL), cetuximab (170 μg/mL), or αEGFR-E-P125A (250 μg/mL) for 16 h and harvested; cytoplasmic extracts were prepared, and western blot performed for vimentin or SER39-phosphorylated vimentin. Relative quantification of vimentin-S39-*P*/vimentin using the NIH ImageJ program is shown. (**C**) αEGFR-E-P125A inhibits secretion of MMP2 and shedding of MT1-MMP: MDA-MB-231-4175 cells were plated on matrigel, treated with E-P125A (58 μg/mL), cetuximab (170 μg/mL), or αEGFR-E-P125A (250 μg/mL) for 16 h; supernatants were harvested and analyzed for secreted MMP2 or shed MT1-MMP by ELISA. Details in methods. (**D**) αEGFR-E-P125A treatment reduces β-catenin levels. MDA-MB-231-4175 cells were plated on matrigel, treated with E-P125A (58 μg/mL), cetuximab (170 μg, or αEGFR-E-P125A (250 μg/mL) for 16 h and then harvested; total, cytoplasmic, and nuclear extracts were analyzed by Western blotting. Tubulin was used for cytoplasmic and lamin B for nuclear normalization. The right histogram shows the relative quantification of β-catenin/(tubulin or lamin B) staining using NIH ImageJ software. (**E**) αEGFR-E-P125A inhibits TCF/LEF transcriptional activity. MDA-MB-231 (TCF/LEF Luc Reporter) cells were plated and treated as indicated (Figure 3A). TCF/LEF reporter assay was performed in 96-well plates without (2D) or containing matrigel (3D, shown in the bottom panel) at 16 h. Luciferase activity in treated cells relative to the medium control is indicated.

Extensive cellular projections consistent with sprouting were seen when HUVEC were plated on matrigel during CLS formation. In contrast HUVEC treated with αEGFR-E-P125A did not form CLS, and sprouting was not observed (Figure 3A). Similar to HUVEC, cellular extensions resembling ‘sprouting’ were also seen with MDA-MB-231-4175 undergoing VM in vitro. Both VM and HUVEC ‘sprouting’ was completely inhibited by αEGFR-E-P125A treatment (Figure 3A). Sprouting and tube formation and VM were not inhibited by E-P125A, cetuximab, or the combination of both.

Because of the changes that we observed in HUVEC and TNBC motility, tube formation (VM), and sprouting in vitro, we analyzed the levels of vimentin and SER39-phosphorylated vimentin by western blotting in MDA-MB-231-4175 cells plated in matrigel and treated with PBS, cetuximab, E-P125A, or αEGFR-E-P125A, and harvested 16 h after plating. SER39-phosphorylated vimentin was markedly reduced in αEGFR-E-P125A-treated TNBC cells (Figure 3B). The ratio of SER39-phosphorylated vimentin/vimentin was reduced in αEGFR-E-P125A-treated MDA-MB-231-4175 cells relative to controls (Figure 3B, *p* = 0.0111–0.0200).

### 3.7. αEGFR-E-P125A Reduces Local Elaboration of MMP-2 and MT1-MMP Matrix Metalloproteases

Vimentin is also known to regulate surface translocation of matrix metalloproteases such as MT1-MMP, which play a key role in conferring cancer cell motility and invasive capability [29,38,39]. Local elaboration of matrix metalloproteases including MMP-2, and MT1-MMP (also known as MMP-14) is also necessary for generation of laminin-5-γ2 fragments that are required for the formation of VM channels [29,38,39]. We next examined levels of secreted MMP2 and shed MT1-MMP at 16 h in the supernatant of MDA-MB-231-4175 cultured on matrigel following treatment with E-P125A, cetuximab, or αEGFR-E-P125A We noted a marked reduction in the concentration of soluble MMP2 (Figure 3C, *p* < 0.0001–0.0002), and of shed MT1-MMP (Figure 3C, *p* = 0.0017–0.0062) following αEGFR-E-P125A treatment compared to treatment with E-P125A or cetuximab (Figure 3C). αEGFR-E-P125A therefore reduces vimentin phosphorylation, which may in turn affect the translocation of MT1-MMP and other metalloproteases to the cell surface, leading to decreased migration and invasion by TNBC. Our findings indicate that αEGFR-E-P125A fusion protein treatment may lead to post translational changes in vimentin that inhibit angiogenesis, EMT (epithelial-mesenchymal transition), and EET (epithelium-to-endothelium transition).

### 3.8. αEGFR-E-P125A Inhibits Wnt/β-Catenin Signaling

Wnt/β-catenin signaling is critical for epithelial-mesenchymal transition (EMT) and cell migration and has been implicated in tumor metastatic behavior [40,41,42,43,44,45]. Endostatin inhibits Wnt/β-catenin signaling [46,47]. Wnt signaling is also known to be critical for angiogenesis during retinal development, and endothelial cells including HUVEC express multiple Wnt receptors including Frizzled receptors Fz-4, 5, and 6 [48]. Wnt-1 signaling can activate HUVEC proliferation, through β-catenin stabilization, as well as transcription from TCF-responsive promoters [48]. De Pradip et al. have shown that TNBC VM can be inhibited using pharmacologic inhibitors of β-catenin, which increase axin, leading to β-catenin degradation, or by using inhibitors of GSK3-β, which reduce β-catenin levels [41]. We hypothesized that the αEGFR-E-P125A effects on VM may be associated with a reduction in Wnt-pathway signaling. We therefore tested the αEGFR-E-P125A effects on the expression of total, cytoplasmic, and nuclear β-catenin in vitro using western blot analysis in MDA-MB-231 cells plated in matrigel. αEGFR-E-P125A treatment significantly reduced total, nuclear, and cytoplasmic β-catenin relative to PBS, E-P125A, or cetuximab-treated cells (Figure 3D).

We also tested transcriptional effects on β-catenin-dependent TCF/LEF-reporter activity using MDA-MB-231 cells stably transduced with a TCF/LEF luciferase reporter. Treatment with αEGFR-E-P125A markedly reduced TCF/LEF-dependent luciferase activity relative to PBS, E-P125A or cetuximab-treated controls in both non matrigel (data not shown) and in matrigel culture (Figure 3E, *p* < 0.0001in matrigel culture).

### 3.9. αEGFR-E-P125A Inhibits TNBC Growth and Metastasis

**Figure 4:**
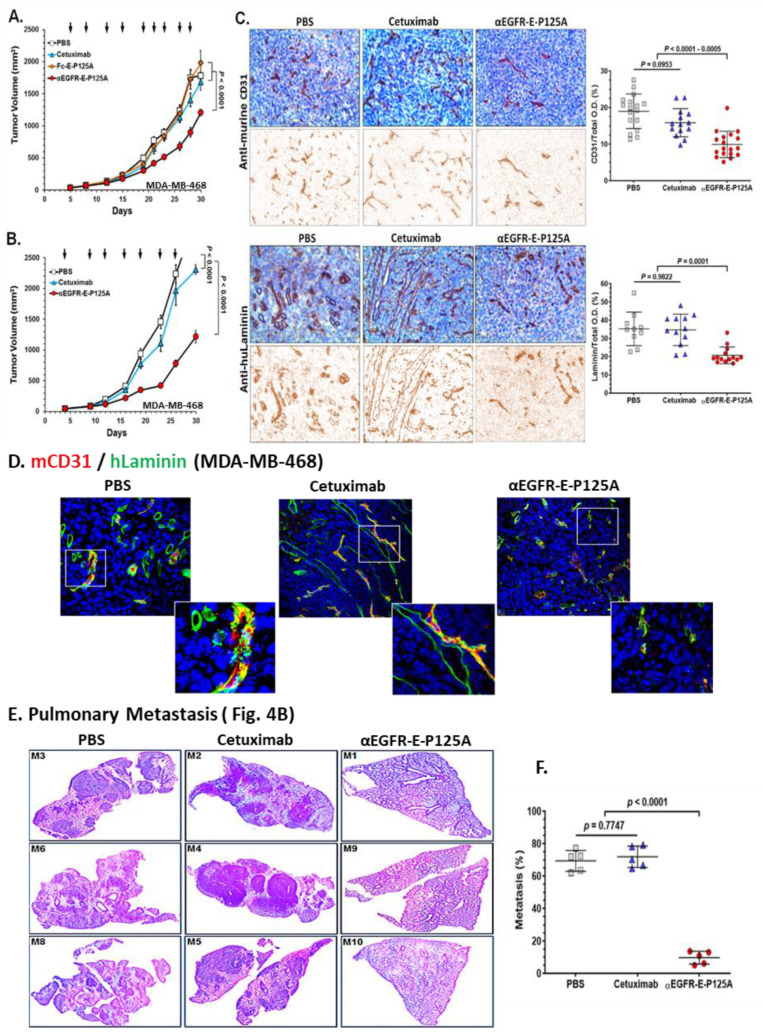
αEGFR-E-P125A effects on angiogenesis, as well as vasculogenic mimicry, tumor growth, and pulmonary metastasis of MDA-MB-468. (**A**) Tumor growth: MDA-MB-468 cells (5 × 10^6^/mouse) were injected into the mammary fat pad (mfp) of female NSG mice (*n* = 8). Starting day 5 post tumor implantation, mice were treated with PBS, equimolar cetuximab (170 μg/injection), Fc-E-P125A (102 μg/injection), or αEGFR-E-P125A (250 μg/injection) at the indicated intervals (black arrows). Tumor size assessed by digital calipers. Data are shown as the mean ± SEM. (**B**). Primary tumor growth and pulmonary metastasis: In a separate experiment from Figure 4A, MDA-MB-468 cells (5 × 10^6^/mouse) were implanted in the mammary fat pad (mfp), and mice were treated intravenously starting day 5 post implantation with PBS, cetuximab (170 μg/injection), or αEGFR-E-P125A (250 μg/injection) at the indicated intervals (black arrows). On day 30, mice were euthanized, and primary mammary tumor growth was measured by calipers (Figure 4B); lungs were harvested for further analysis of pulmonary metastases (Figure 4E,F). Data are shown as the mean ± SEM. (**C**) Immunohistochemical staining of MDA-MB-468 tumor for angiogenesis and VM. Primary tumor sections from Figure 4B stained for murine CD31 (mCD31, the upper panel) or for human laminin (the bottom panel) and counterstained with hematoxylin on day 30. Representative sections from a tumor selected for ‘average’ size are shown. Relative positive optical density/total optical density of the immunohistochemical staining was analyzed using NIH ImageJ software for individual mouse lung sections and represented as yellow squares (PBS treated), blue triangles (cetuximab treated) or red circles (αEGFR-E-P125A treated). Details in Methods. (**D**) Immunofluorescence staining for mCD31 (red) and anti-human laminin (green) day 30 post tumor implantation. Vessels staining green only for human laminin correspond to areas of VM, while vessels staining red mixed with green represent mCD31+/huLaminin+ ‘mosaic ‘ vessels. Details in Methods. (**E**,**F**). αEGFR IgG1-huEndo-P125A inhibits pulmonary metastasis: (**E**) Formalin-fixed paraffin-embedded lung tissues from representative mice from the 10 mice/treatment group with near ‘average’ growth of a primary mfp implanted MDA-MB-468, stained with hematoxylin and eosin. (**F**) Metastatic burden in the lung sections (5 out of 10 mice) for the experiment represented in Figure 4B,C. Relative percent metastasis containing area/total lung area in individual murine lung sections was analyzed using NIH ImageJ software, and relative percent metastases for individual mouse lung sections are represented as yellow squares (PBS treated), blue triangles (cetuximab treated) or red circles (αEGFR-E-P125A treated). Details in Methods.

Since αEGFR-E-P125A and Fc-E-P125A both inhibited angiogenesis and TNBC VM in vitro, we next tested drug efficacy in vivo against TNBC xenografts. To assay effects on angiogenesis, we stained TNBC xenograft tumor sections for murine CD31 to identify murine vessels and stained excised tissues for human laminin to outline VM channels [2,49]. We compared the effects of treatment on orthotopically implanted MDA-MB-468 tumors in NSG mice (female, 10 mice per group). αEGFR-E-P125A significantly decreased primary tumor growth relative to either cetuximab or Fc-E-P125A-treated controls (Figure 4A, *p* < 0.0001). Therefore, targeting of EGFR increased in vivo efficacy against EGFR + TNBC xenografts.

In a separate experiment, MDA-MB-468 cells (5 × 10^6^/mouse) were implanted in the mammary fat pad (mfp), and mice (female, 10 mice per group) were treated intravenously starting day 5 post implantation with PBS, equimolar cetuximab (170 μg/injection), or αEGFR-E-P125A (250 μg/injection) at the indicated intervals (black arrows). αEGFR-E-P125A decreased primary tumor growth relative to cetuximab or PBS-treated mice (Figure 4A,B, *p* < 0.0001).

Angiogenesis was detected in tumor tissue immunohistochemically with anti-murine CD31 to host endothelial cells, while the contribution of human TNBC tumor cells to VM was detected using anti-human laminin as described [2,49,50,51]. We noted a significant decrease in murine CD31+ blood vessels in the tumor bed of αEGFR-E-P125A-treated mice as assayed by immunohistochemical staining (IHC) (Figure 4C). IHC staining for human laminin (huLaminin) identified clear VM channels, which stained longitudinally or in cross section. No human laminin staining was observed in normal murine tissues such as lung, brain, muscle, kidney, or liver in the absence of tumor (data not shown). We noted a marked decrease in huLaminin+ VM channels in αEGFR-E-P125A-treated mice by IHC (Figure 4C) (*p* < 0001 compared to PBS-treated control or cetuximab-treated mice).

Using immunofluorescence, we stained MDA-MB-468 TNBC tumor sections for both mCD31 to outline murine vessels and huLaminin to simultaneously label VM channels (Figure 4D) [2,49,50,51]. PBS- or cetuximab-treated sections demonstrated abundant human Laminin+ tube-like structures consistent with VM (Figure 4D). Red mCD31+ blood vessels frequently overlapped with or were contained within green huLaminin+ tube-like structures, consistent with ‘mosaic’ vessel formation. αEGFR-E-P125A significantly reduced both mCD31+ vessels and huLaminin+ VM and ‘mosaic’ vessel staining compared to the effects of either PBS or cetuximab (Figure 4D). Furthermore, we noted that nearly all red mCD31+ vessels in control PBS- or cetuximab-treated mice were embedded within or completely overlapped huLaminin+ VM channels (green). αEGFR-E-P125A-treated mice showed markedly decreased mCD31+ vasculature as well as VM channels. The presence of overlapping VM and mCD31 ‘mosaic’ vascular structures was also markedly reduced (Figure 4D). While cetuximab reduced angiogenesis modestly, αEGFR-E-P125A reduced both mCD31+ staining and huLaminin+ VM networks compared to either cetuximab- or PBS-treated animals (Figure 4C,D).

In addition to the effects on the primary tumor, we examined the lungs of five randomly selected mice from each group for metastatic spread. We noted a decrease in the size and number of metastases in αEGFR-E-P125A-treated mice compared to untreated or cetuximab-treated controls (representative day 30 lung sections are shown in Figure 4E). αEGFR-E-P125A significantly inhibited the formation of pulmonary metastasis on day 30 relative to PBS- or cetuximab-treated mice (*p* < 0.0001), as determined using NIH ImageJ quantitation of the image area involved in lung metastases (Figure 4F).

**Figure 5:**
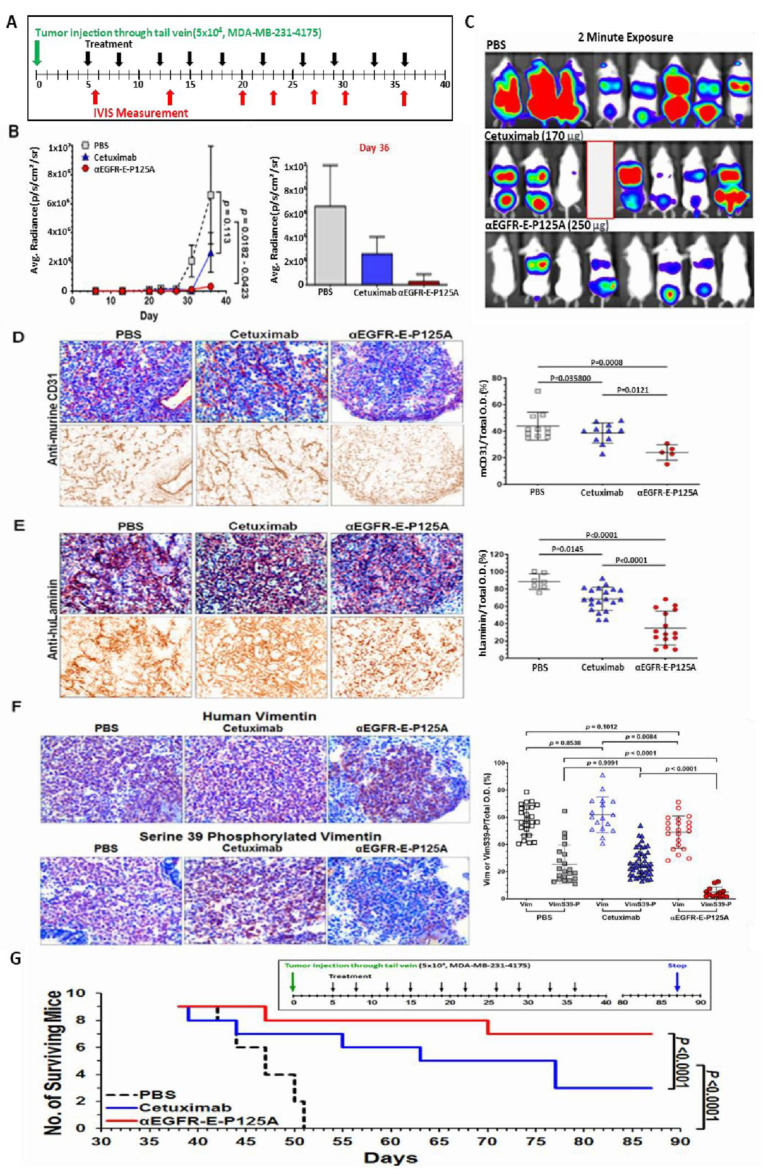
(**A**–**F**) αEGFR IgG1-huEndo-P125A inhibits pulmonary tropic MDA-MB-231-4175 lung metastasis. (**A**) Pulmonary metastasis: 5 × 10^4^ MDA-MB-231-4175 cells were injected via the tail vein (green arrow). Mice (*n* = 8) were treated starting day 5 with PBS, cetuximab (170 µg/injection), or αEGFR-E-P125A (250 µg/injection) 2×/week (black arrows, 9 injections). Metastases were monitored by IVIS at intervals indicated by red arrows. Experiment terminated on day 36. A single mouse was lost in the cetuximab group on day 35 (a red blank in Figure 5C). (**B**,**C**) Pulmonary average photon flux graph (**B**) and relative photon flux/2-min exposure on day 36 (**C**) The αEGFR-E-P125A-treated group showed reduced total body tumor burden and lung metastasis. Average photon flux on day 36 is shown in a graph. Data shown as the mean ± SEM. (**D**,**E**) αEGFR-E-P125A inhibits mCD31+ angiogenesis (D) and huLaminin+ TNBC VM (E) of MDA-MB-231-4175. Immunohistochemical staining of tumor sections on day 36 from mice in Figure 5B and Figure 6C. Cryosections from representative treated mice were stained with anti-murine CD31 (**D**) or anti-human laminin antibody (**E**) and counterstained with hematoxylin. Relative positive optical density/total optical density of the immunohistochemical staining was analyzed using NIH ImageJ. Each dot represents an immunohistochemically stained cryosection from a tumor selected for near ‘average’ photon flux intensity. Details in Methods. (**F**) αEGFR-E-P125A inhibits vimentin-SER39 phosphorylation: On day 36, lung sections containing metastatic MDA-MB-231-4175 from sacrificed mice (Figure 5B,C) were analyzed by IHC. Cryosections from mice with near average relative photon flux intensity stained for human vimentin (brown, Vim, **the upper panel**) or human vimentin phosphorylated on serine 39 (brown, VimS39-P, **bottom panel**). Hematoxylin was used for nuclear counter-staining. Representative tumor cryosections from mice treated with PBS, cetuximab, or αEGFR-E-P125A are presented. Relative vimentin+ or SER39 vimentin+ staining optical density/total optical density of IHC staining was quantified using NIH ImageJ. (**G**) Survival of mice following αEGFR-E-P125A treatment. MDA-MB-231-4175 cells (5 × 10^4^) were injected via the tail vein and survival was measured. Nine mice/group were treated starting day 5 with PBS, cetuximab (68 µg/injection), or αEGFR-E-P125A (100 µg/injection) 2×/week (10 injections) per schedule shown above the survival curve. Survival was analyzed using the survival analyses and one-way ANOVA test (GraphPad Prism 7.03). Green arrow, tumor inoculation; black arrow: treatment administration ( PBS, cetuximab, or αEGFR-E-P125A as indicated), blue arrow: experiment termination.

We next tested the effects of αEGFR-E-P125A on tumor metastasis using the highly metastatic lung-tropic MDA-MB-231 variant line, MDA-MB-231-4175,with lower but detectable levels of EGFR expression and a high proclivity to form lung metastases [21]. This model was tested for the ability to rapidly form lung metastases following direct intravenous injection, rather than metastases from primary tumor. Cells were directly injected via the tail vein, and equimolar cetuximab, or αEGFR-E-P125A treatment was initiated day 5 post TNBC injection and continued 2×/weekly for nine doses (Figure 5A). Metastatic spread was quantified using an in vivo imaging system (IVIS) on day 36 (Figure 5B,C). αEGFR-E-P125A significantly inhibited pulmonary metastasis formation on day 36 relative to PBS- or cetuximab-treated mice (PBS, *p* = 0.0182; cetuximab, *p* = 0.0423), as quantitated by IVIS (Figure 5B,C).

We stained lung sections from mice following *iv* injection of MDA-MB-231-4175 (from Figure 5B). αEGFR-E-P125A treatment of mice markedly reduced metastases (Figure 5B). αEGFR-E-P125A treatment reduced mCD31+angiogenesis on day 36 compared to PBS or cetuximab treatment (Figure 5D, PBS: *p* = 0.0008, cetuximab: *p* = 0.0121). Extensive staining of huLaminin+ networks was seen in PBS-treated control mice. Cetuximab-treated mice demonstrated a decrease in huLaminin+ VM networks relative to PBS treatment (Figure 5E. *p* = 0.0145). Treatment with αEGFR-E-P125A further decreased huLaminin+ staining density relative to PBS (*p* < 0.0001) or cetuximab treatment (*p* < 0.0001) (Figure 5E).

Due to the observed effects on Ser39 phosphorylation of vimentin in vitro, we also investigated the αEGFR-E-P125A effects on levels of vimentin and vimentin-SER39 phosphorylation in vivo. Lung cryosections of MDA-MB-231-4175 challenged mice were stained for human vimentin and for SER39-phosphorylated vimentin (Figure 5F). High levels of vimentin were observed in PBS- or cetuximab-treated tumor sections, but lower levels of vimentin were seen in αEGFR-E-P125A-treated tumor sections (Figure 5F). However, markedly lower levels of SER39-phosphorylated vimentin were noted in sections from αEGFR-E-P125A-treated mice (Figure 5F, *p* < 0.0001).

To determine whether the observed decrease in metastases would confer a long-term survival advantage following cessation of therapy, MDA-MB-231-4175 cells (5 × 10^4^) were injected via the tail vein and survival was measured. Nine mice/group were treated starting on day 5 with PBS, cetuximab, or αEGFR-E-P125A 2×/week (10 injections) per the schedule shown above the survival curve. Treatment was stopped following a total of ten injections. Survival was analyzed using the survival analyses and one-way ANOVA test (GraphPad Prism 7.03). Mice treated with αEGFR-E-P125A showed significantly improved survival relative to cetuximab- or PBS-treated controls (*p* < 0.0001) (Figure 5G). At 87 days, 77.8% of αEGFR-E-P125A-treated mice survived, compared to 33.3% for cetuximab and 0% survival for PBS treated mice (*p* < 0.0001). Therefore, αEGFR-E-P125A treatment resulted in prolonged survival of treated mice despite treatment cessation.

**Figure 6:**
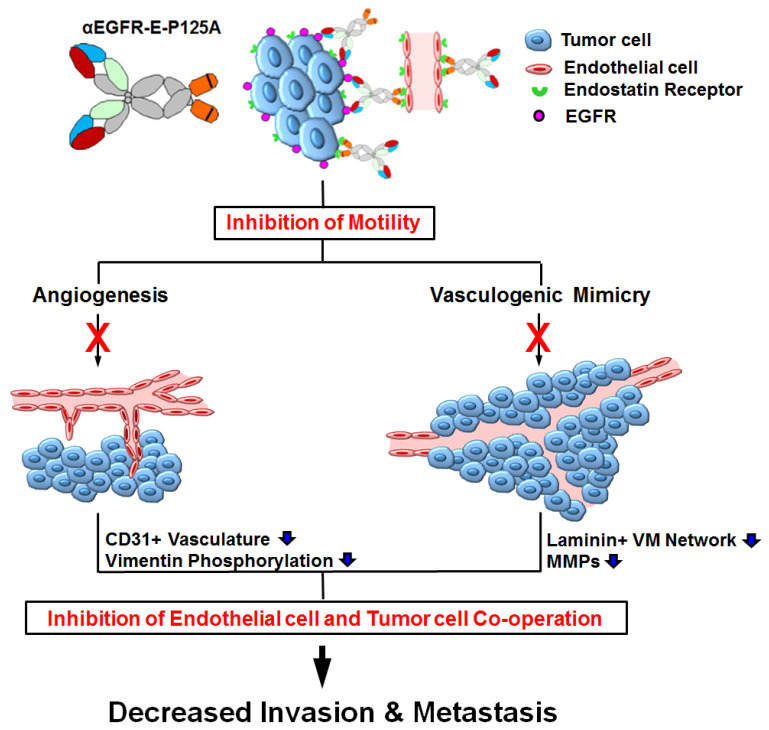
αEGFR-E-P125A effects on angiogenesis and VM lead to decreased tumor growth and metastasis: αEGFR-E-P125A reduces TNBC motility and migration, leading to decreased circulating tumor cells. Assembly of tumor blood vessels (angiogenesis) by endothelial precursors and tumor cell-lined vascular channels (VM) are both inhibited, reducing tumor growth and metastasis.

In summary, αEGFR-E-P125A inhibited TNBC tumor cell VM and angiogenesis, leading to decreased TNBC tumor growth and metastasis (see model, Figure 6).

## 4. Discussion

Development of vasculature is essential for neoplastic progression and metastasis. In addition to canonical angiogenesis, cancer cells can form channels lined by tumor cells that can anastomose with existing blood vessels, a phenomenon known as vasculogenic mimicry (VM) (1). VM is highly prevalent in TNBC. TNBC cells demonstrate significant plasticity and are capable of forming vascular networks that are perfused in the absence of lined endothelial cells. VM is thought to be able to anastomose with the normal circulation in order to supply nutrients to growing tumors. VM may contribute to TNBC aggressiveness and has been linked to resistance against anti-angiogenic agents [7]. TNBC or basal-like subtypes of breast cancer frequently metastasize, resulting in poor clinical outcomes. VM has been reported to be highly prevalent and more frequently seen in the TNBC relative to luminal or HER2+ positive breast cancer [52].

Here, we constructed an αEGFR-E-P125A-targeting EGFR using an IgG1 backbone. In this study, we demonstrate the ability of αEGFR-E-P125A to bind EGFR and deliver a dimeric mutant endostatin ‘payload’ that efficiently inhibits endothelial angiogenesis, TNBC VM, and retains the ability to mediate ADCC. αEGFR-E-P125A is more effective at inhibiting VM in vitro than E-P125A, cetuximab, or the combination. αEGFR-E-P125A inhibited tumor cell motility in vitro and reduced primary TNBC growth and TNBC metastases in two highly metastatic TNBC xenograft models, as well as significantly improved the survival of treated tumor-bearing mice compared to cetuximab-treated controls.

Endostatin, a 22 kD fragment of collagen XVIII, is a naturally occurring inhibitor of angiogenesis in vivo [13,14]. Despite promising preclinical studies, endostatin failed to demonstrate significant anti-tumor activity in human Phase I/II trials [53,54]. The postulated reasons for reduced efficacy include (i) clinically tested endostatin expressed in yeast lacked N-terminal amino acids essential for Zn-binding [55]; (ii) limited tumor exposure due to poor delivery and short half-life. Modified endostatin with reinstated N-terminal amino acids and increased stability was approved following positive Phase III NSCLC trials in China, and efficacy has been reported in breast cancer and other trials [56]. Differences in αEGFR-E-P125A relative to clinically tested endostatin include (i) E-P125A in the fusion contains N-terminal Zn binding amino acids and has superior anti-angiogenic activity relative to wild type endostatin [17], (ii) the targeting anti-EGFR antibody delivers dimeric E-P125A, (iii) neither monomeric endostatin nor E-P125A demonstrates VM and TNBC migration inhibition at comparable concentrations, suggesting unique properties related to the dimeric presentation of E-P125A.

In our prior studies, a prototypic αHER2 IgG3-huEndo-P125A fusion protein demonstrated 40–50-fold longer half-life in mice compared to endostatin, superior anti-angiogenic efficacy, and enhanced anti-tumor activity in vivo [15,16]. αHER2 IgG3-huEndo-P125A demonstrated greater efficacy against breast cancer xenografts compared to an αHER2 fusion with wild type endostatin [16]. However, in that study, we did not look for effects on VM. This was in part due to the absence of evidence for VM in vitro in the breast cancer model employed (SK-BR-3). More recent reports note that VM can be induced in vitro in matrigel for SK-BR-3, using additional angiogenic and growth factors. SK-BR-3 cells do readily form mosaic vessels when cultured in the presence of HUVEC (data not published), and evidence for VM in this aggressive model in vivo has also been reported [57]. Whether inhibition of SK-BR-3 breast cancer xenograft growth seen by αHER2 IgG3-huEndo-P125A was also associated with a reduction in VM as well as angiogenesis remains to be studied.

Wnt/β-catenin mediated signaling has been found to play an important role in embryogenesis and also in a variety of processes leading to cancer-related morbidity and mortality. Deregulation of the Wnt pathway has been demonstrated in breast cancer [41,58,59]. The Wnt pathway may be deregulated through expression of Wnt ligands or through inactivation of β-catenin regulatory pathways such as APC inactivation. The Wnt pathway has been implicated in the regulation of VM in triple negative breast cancer. Partial reversal of the VM was accomplished by those investigators using inhibitors of the PI3K pathway, pharmacologic inhibition of β-catenin [41], and in our study by using an antibody–endostatin fusion. In each case, vasculogenic mimicry as well as angiogenesis was inhibited, leading to impaired tumor growth and improved survival of tumor-bearing animals. Further testing in additional xenograft and PDX models may be required to validate observations seen in the current xenograft studies. αEGFR-E-P125A reduced levels of cytoplasmic, nuclear, and total β-catenin. TNBC containing a TCF-LEF reporter demonstrated a reduction in transcription, consistent with reduced Wnt signaling. Wnt/β-catenin signaling has been implicated in VM, EMT, cancer stem cell-like transformation, and impaired anti-tumor immunity [40,41,43]. In addition, the Wnt/β-catenin pathway has been directly linked to the regulation of vasculogenic mimicry in TNBC [52]. Potential mechanisms underlying the therapeutic effects of αEGFR-E-P125A include the induction of β-catenin degradation, leading to reduced Wnt pathway signaling, reduction in SER39 phosphorylated and overall vimentin levels, in turn preventing local elaboration of MMP2 and MT1-MMP further limiting invasive behavior.

Vimentin is a highly abundant and conserved type III intermediate filament protein. It generally is expressed at high levels in cells of mesenchymal origin. Intermediate filament networks are subject to regulation by a variety of kinases and phosphatases. Multiple phosphorylation sites have been identified. Vimentin can be cleaved by proteases at multiple sites, resulting in lower molecular weight fragments that are important for angiogenesis and cell movement. Vimentin phosphorylation also plays a critical role in regulating TNBC motility, EMT, and in localization of MMP during invadopodia formation [2,36,38,60,61]. Levels of vimentin in MDA-MB-231-4175 cells and MDA-MB-231 were considerably higher based on Western blot and immunohistochemistry in MDA-MB-231-4175 cells and MDA-MB231 than in MDA-MB468, which retained epithelial characteristics and did not undergo similar mesenchymal differentiation (data not shown) Epithelial to endothelial transition (EET) shares many phenotypic characteristics of EMT, and hypoxia can promote VM in renal cell and non-small cell carcinoma [31] and is also associated with TNBC EET, EMT, and in turn, VM [31,32,33,34]. Expression of EMT-associated markers including vimentin has been linked to poor prognosis in triple negative breast cancer [30]. In addition, vimentin and specifically vimentin post translational modification by phosphorylation play a key role in angiogenesis, endothelial cell sprouting, MT1-MMP translocation, and endothelial cell invasion [38]. We observed inhibition of ‘sprouting’ in both HUVEC and of TNBC forming tubes in matrigel by αEGFR-E-P125A. Endothelial sprouting has been linked to S39 vimentin phosphorylation. Because of the significant effects observed on TNBC VM and high baseline levels of vimentin expression, we analyzed vimentin and S39-phosphorylated vimentin levels in MDA-MB-231-4175 cells undergoing treatment in vitro or in vivo with αEGFR-E-P125A fusion and in both instances demonstrated a significant reduction in S39 vimentin phosphorylation and reduced VM and metastases. αEGFR-E-P125A reduced the levels of SER39-phosphorylated vimentin in TNBC cells cultured in matrigel and in tumor deposits in vivo. αEGFR-E-P125A may inhibit EMT, VM, and invasiveness in part through effects on vimentin phosphorylation and function. Our findings indicate that αEGFR-E-P125A treatment may lead to post translational changes in vimentin that impact angiogenesis, EMT, and EET. αEGFR-E-P125A may also affect integrin-mediated cell attachment, as well as the activity of focal adhesions. Vimentin is also known to play a role in the regulation of FAK (focal adhesion kinase) and endothelial sprouting [38], reducing angiogenesis.

Endostatin binds several integrins including αvβ3 [62]. Camorani et al. reported that an anti-EGFR directed aptamer, but not erlotinib or cetuximab, prevented VM by MDA-MB-231 through disruption of the integrin αvβ3 interaction with EGFR and inhibited tumor growth [63]. Whether αEGFR-E-P125A similarly disrupts EGFR-integrin interactions is currently under study. Although VM inhibition was seen in vitro with Fc-E-P125A, which does not target EGFR (Supplementary Figure S2C), αEGFR-E-P125A demonstrated greater efficacy against EGFR+ MDA-MB-468 xenografts than Fc-E-P125A in vivo (Figure 4A), suggesting that EGFR targeting is needed for optimal anti-tumor efficacy. Additional investigation of signaling events following αEGFR-E-P125A treatment of both HUVEC and TNBC may unmask shared affected pathways that are fundamental to both VM and angiogenesis.

VM may contribute to resistance against anti-angiogenic agents. Sun et al. reported that VEGF blockade with sunitinib in TNBC xenografts promoted an increase in VM, followed by rebound formation of endothelium-dependent vessels following sunitinib discontinuation [5]. Whether ‘rebound endothelialization’ is preceded by formation of ‘mosaic’ endothelial: tumor cell lined vessels is not known. Mosaic vessel formation was inhibited in vitro and also markedly reduced in vivo in response to αEGFR-E-P125A treatment (Figure 2). It would be interesting to test the effects of αEGFR-E-P125A delivered along with or following sunitinib or bevacizumab to see whether ‘rebound endothelialization’ could be prevented due to inhibition of VM [7].

In addition to enhanced αEGFR-E-P125A inhibitory effects on TNBC VM compared to cetuximab, E-P125A or a combination of the two agents, αEGFR-E-P125A fusion also retained the ability to mediate ADCC. ADCC activity appears to require the presence of the anti-EGFR domain. The retained ability to mediate ADCC may further contribute to an anti-tumor immune response, an important property since TNBC respond to checkpoint inhibition [64].

In summary, αEGFR-E-P125A is an exciting new therapeutic modality that inhibits key physiologic processes linked to TNBC invasiveness, motility, and metastasis, including angiogenesis and VM. VM and angiogenesis inhibition is associated with a reduction in vimentin phosphorylation and/or Wnt/β-catenin signaling. Combined inhibition of angiogenesis, VM, tumor cell migration, and invasive behavior is a potent strategy for inhibiting TNBC growth and metastasis (Figure 6).

## Data Availability

The data presented in this study are available in the article and/or supplementary material presented here. Any needed clarifications and/or additional supporting data is available upon request tfrom the corresponding author (J.D.R.).

## Supplementary Materials

The following are available online at https://www.mdpi.com/article/10.3390/cells10112904/s1, Figure S1. Western blot and flow cytometry analysis of αEGFR IgG1-huEndo-P125A fusion protein. Figure S2. Binding and ADCC activity of αEGFR-E--P125A to TNBC cells. Figure S3. Scratch Wound Assay of TNBC and HUVEC Migration. Table S1. Materials list.
