# Supplementary material for: Inhibition of Vasculogenic Mimicry and Angiogenesis by an Anti-EGFR IgG1-Human Endostatin-P125A Fusion Protein Reduces Triple Negative Breast Cancer Metastases
Source: Cells. 2021 Oct 27;10(11):2904. doi: 10.3390/cells10112904 (PMC8616280; doi:10.3390/cells10112904)
Supplement: Supplementary file 1 [file cells-10-02904-s001.zip › cells-1357653-SI.pdf]

Fig.S1

A. Western blot analysis of αEGFR-E-P125A fusion protein

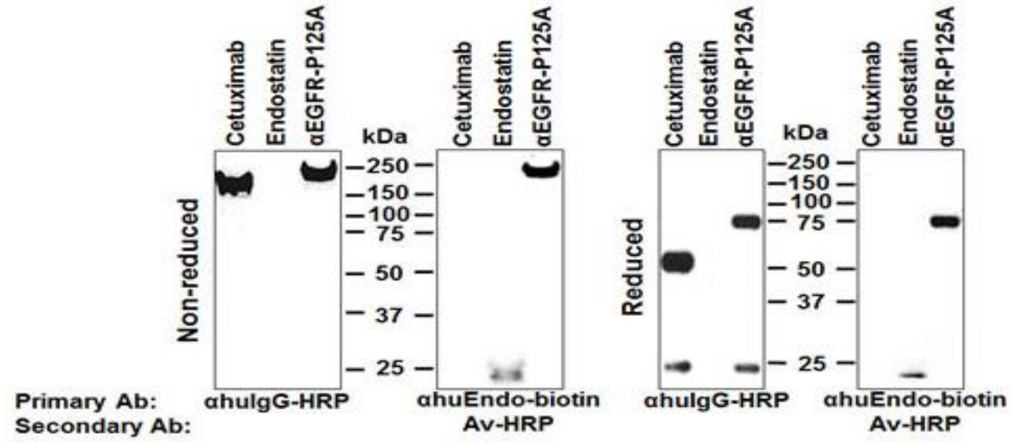

B. Binding of αEGFR-E-P125A using anti-human IgG

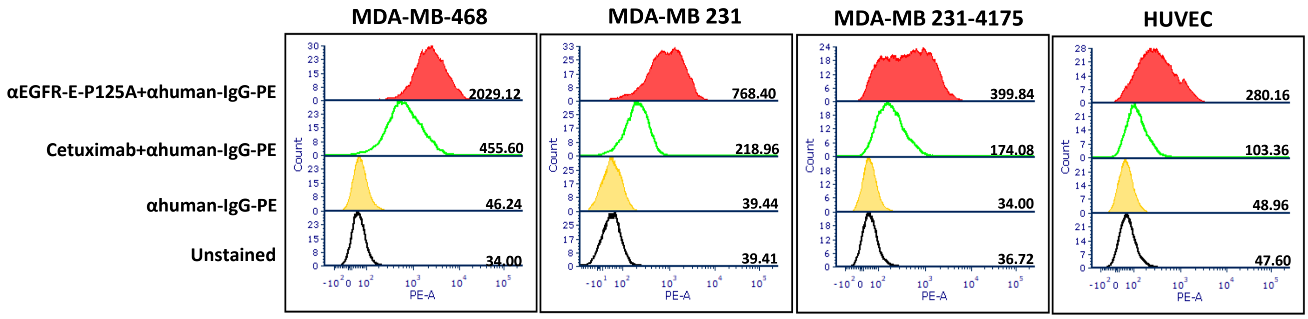

C. Binding of αEGFR-E-P125A using biotinylated anti-human Endostatin

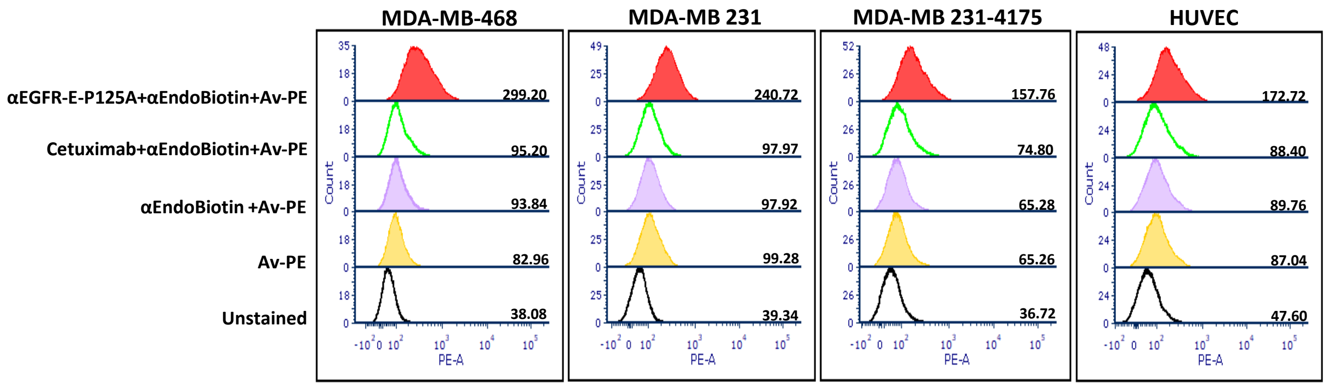

Fig. S1. A. Western blot analysis of αEGFR IgG1-huEndo-P125A fusion protein. αEGFR IgG1-huEndo-P125A (αEGFR-P125A), cetuximab, and endostatin resolved under nonreducing and reducing conditions. To detect fused huEndo-P125A, biotinylated anti-human endostatin antibody was used as a primary, and avidin conjugated horseradish peroxidase as a secondary antibody. To detect human IgG1, goat anti-human IgG conjugated with horseradish peroxidase was used.

B-C. Binding of anti-EGFR IgG1-huEndoP125A to EGFR (B), and recognition by anti-human endostatin (C). HUVEC and MDA-MB-231 were incubated with αEGFR IgG1-huEndo-P125A (αEGFR IgG1-P125A, filled with red), or cetuximab (green line). Bound fusion proteins were identified with PE conjugated anti-human IgG (B) or with biotinylated anti-human endostatin antibody and avidin-PE conjugate (C).

Fig.S2

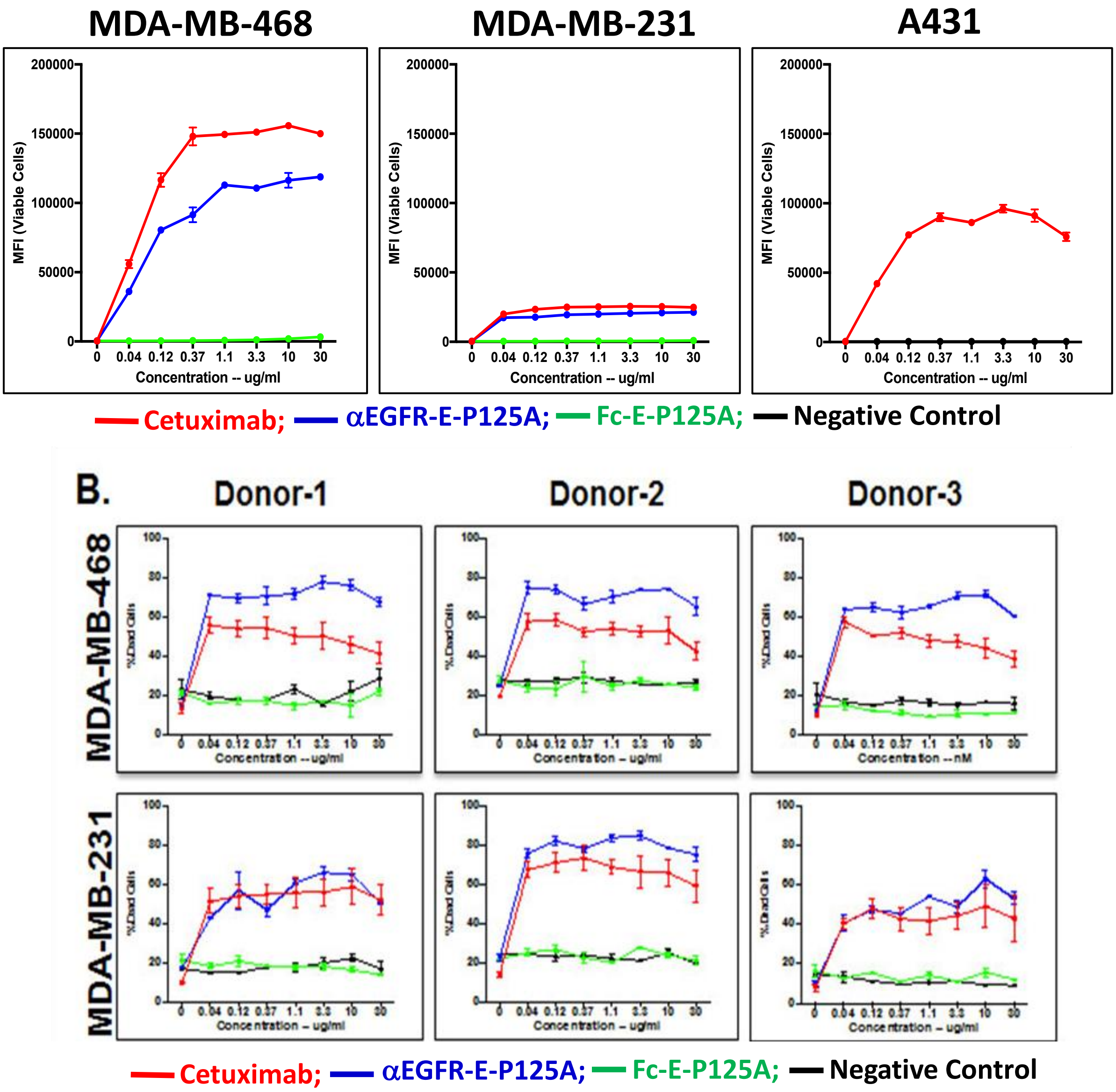
MDA-MB-468
MDA-MB-231
A431
MFI (Viable Cells)
Concentration -- ug/ml
Cetuximab; αEGFR-E-P125A; Fc-E-P125A; Negative Control
B.
Donor-1
Donor-2
Donor-3
MDA-MB-468
MDA-MB-231
Cetuximab; αEGFR-E-P125A; Fc-E-P125A; Negative Control

Fig S2. A. Binding of αEGFR-E-P125A to TNBC target cells. MDA-MB-468 and MDA-MB-231 TNBC were tested. A431 cells were used as an EGFR+ positive control for cetuximab binding. Cells were incubated with cetuximab, αEGFR-E-P125A, Fc-E-P125Aor a negative control antibody (anti-human IgG) for binding to EGFR at 4°C for 45 min, stained with anti-human IgG conjugated with AlexaFluor 488, resuspended and analyzed in an Attune NxT Flow Cytometer. Mean fluorescence intensity (MFI) and binding data were analyzed with Graphpad Prism software.

B. ADCC activity. ADCC activity was determined using a FACS based ADCC assay. Fresh PBMCs purified from normal blood donors served as effector cells. CFSE labeled target cells (~~MDA-MB-231,~~ MDA-MB-468 and MDA-MB-231 ~~, and A431~~) and PBMC were used at a ratio of 1:50. Indicated antibodies or fusion proteins, and target-effector cell mixture were incubated in triplicate at 37°C for 4 hrs. Non-specific IgG1 and Fc-E-P125A were used as negative controls. Cells from the wells were transferred to a FACS tubes containing 0.5% BSA in PBS (pH 7.4) and propidium iodide (PI, 2μg/ml), and cell killing analyzed by flow cytometry (iQue Screener, Intellicyt or BD FACScan, BD Biosciences). ADCC was assessed by measuring frequency of dead target cells (CFSE+/PI+)/ total target cells in the sample (CFSE+/PI+ and CFSE+/PI-). Co-ordinates noted in X-axis denote antibody or fusion concentration ranging from 0.04 to 30 μg/ml while % dead cells are noted in the Y-axis. Details are in Methods

Fig.S3

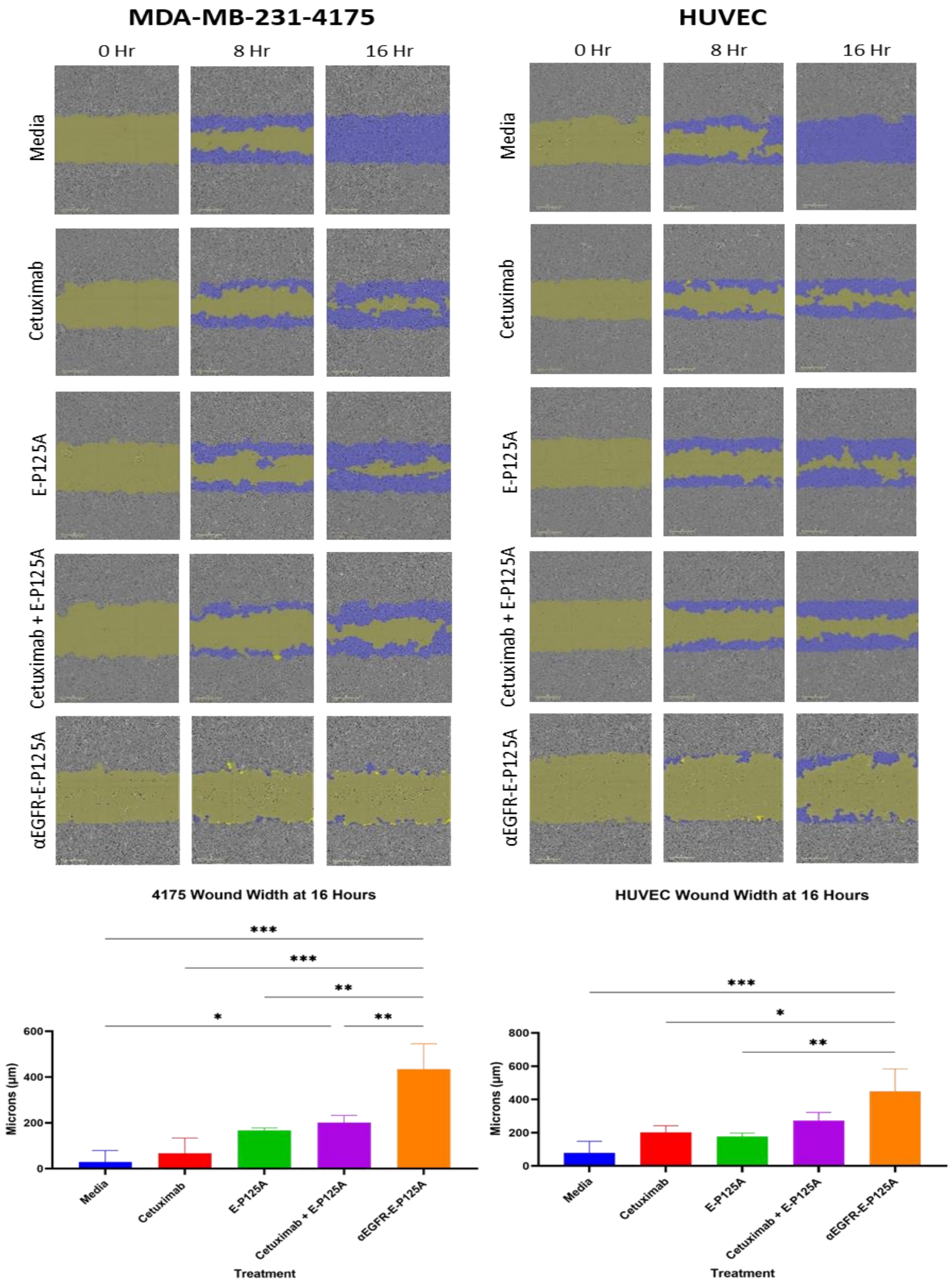
MDA-MB-231-4175
HUVEC
0 Hr
8 Hr
16 Hr
Media
Cetuximab
E-P125A
Cetuximab + E-P125A
αEGFR-E-P125A
4175 Wound Width at 16 Hours
HUVEC Wound Width at 16 Hours
Microns (µm)
Treatment
***
**
*
Media
Cetuximab
E-P125A
Cetuximab + E-P125A
αEGFR-E-P125A
0
200
400
600
800

Fig S3.

Scratch wound migration assay of MDA-MB-231-4175 and HUVEC cells. Effects of αEGFR-E-P125A on tumor and endothelial cell migration were tested by scratching MDA-MB-231-4175 and HUVEC TNBC cell layers using the WoundMaker™. Cells were conditioned with Media, Cetuximab, E-P125A, a combination of Cetuximab and E-P125A, or αEGFR-E-P125A. The wounded cells were put into the IncuCyte ZOOM® for 16 hours to monitor the migration of the cells (scratch wound mask shown in yellow represents changing wound size over time, and is overlaid with the initial scratch wound mask depicted in blue). Compared to the controls, both MDA-MB-231-4175 and HUVEC wound closures were maximally inhibited by αEGFR-E-P125A treatment (bottom row of images). Bar graphs represent the final wound widths computed at the 16-hour time point and were analyzed using one-way ANOVA tests (n=3).

Table.S1 Material List

Supplementary Table 1: Materials and Sources

Name	Host Species	Host Reactivity	Type	Company	Address	Cat #	Dilution
**Cell Lines**							
HUVEC				ATCC	Manassas, VA	PCS-100-010	
MDA-MB-231				ATCC	Manassas, VA	HTB-26	
MDA-MB-468				ATCC	Manassas, VA	HTB-132	
CHO				ATCC	Manassas, VA	CRL-12023	
**Media**							
EGM-2 BulletKit				Lonza	Walkersville, MD	CC-3162	
FBS				Gibco-BRL	Frederic, MD	26140079	
Penicillin-Streptomycin				Gibco-BRL	Frederic, MD	15140122	
RPMI 1640				Gibco-BRL	Frederic, MD	11875093	
**Materials**							
Cell Tracker Red CMTPX				Invitrogen	Frederic, MA	C34552	0.5 μg/ml
Cell Tracker Green CMFDA				Invitrogen	Frederic, MA	C7025	0.5 μg/ml
Cell recovery solution				Corning	Bedford, MA	354253	
Cellomics				Thermofisher Scientific	Waltham, MA	Cellomics Arrayscan VTI	
Cignal lenti TCF/LEF-1 Luciferase reporter				Qigen	Germantown, MD	CLS-018L	
CIM-16 Plates				ACEA	San Diego, CA	5665817001	
Endostatin (Human)				Genscript	Piscataway, NJ	Z02533	
Fluoro-gel II with DAPI				Electron Microscopy Sciences	Hatfield, PA	17985	
Hematoxylin				Vector Laboratories	Burlingame, CA	H3401	
Leica Confocal microscopy				Leica	Buffalo Grove, IL	LAS AF	
Luciferin				PerkinElmer	Waltham, MA	122799	
Matrigel				Corning	Bedford, MA	356235	
MT1-MMP ELISA kit				Abcam	San Francisco, CA	ab197747	
MMP2 ELISA kit				Abcam	San Francisco, CA	ab100606	
NSG				Jackson Laboratory	Bar Harbor, ME	OO5557	
OCT compound				Sakura	Torrance, CA	0000582-03	
Sealant				Biotium	fremont, CA	23005	
xCelligence				ACEA	San Diego, CA		
**Primary Antibodies**							
α-Tubulin	Rabbit	Human	Monoclonal	CST(cell signaling Technology)	Danvers, MA	2125	1:1000
β-Catenin	Mouse	Human	Monoclonal	BD Bioscience	Sparks, MD	610153	1:2000
CD31 conjugated with biotin	Rat	Mouse	Monoclonal	BD Bioscience	Sparks, MD	553371	1:200
Cortactin	Rabbit	Human	Monoclonal	Abcam	San Francisco, CA	ab81208	1:1000
Cyclin D1	Rabbit	Human	Monoclonal	CST(cell signaling Technology)	Danvers, MA	2978	1:1000
Endostatin conjugated with biotin	Goat	Human	Polyclonal	R&D system	Minneapolis, MN	BAF1098	1:10000
Lamin B1	Rabbit	Human	Monoclonal	CST(cell signaling Technology)	Danvers, MA	13435	1:1000
Laminin	Rabbit	Human	Polyclonal	Abcam	San Francisco, CA	ab23753	1:200
TKS5 (WB)	Rabbit	Human	Polyclonal	CST(cell signaling Technology)	Danvers, MA	16619	1:1000
TKS5 (IHC)	Mouse	Human	Monoclonal	Sigma-Millipore	St.Louis, MO	MABT336	1:50
Vimentin (IHC)	Rabbit	Human	Monoclonal	Abcam	San Francisco, CA	ab133260	1:200
Vimentin(S39) phosphorylated	Rabbit	Human	Polyclonal	CST(cell signaling Technology)	Danvers, MA	13614	1:1000 (WB), 1:50 (IHC)
Vimentin (WB)	Rabbit	Human	Monoclonal	CST(cell signaling Technology)	Danvers, MA	5741	1:1000
**Secondary Antibodies**							
anti-human IgG-FITC	Goat	Human	polyclonal	Sigma-Millipore	St.Louis, MO	F0132	1:100
anti-human IgG-HRP	Goat	Human	polyclonal	Sigma-Millipore	St.Louis, MO	A0293	1:20000
anti-rabbit IgG-alexa 488	Goat	Rabbit	polyclonal	Invitrogen	Frederic, MA	A-11034	1:1000
anti-mouse IgG-alexa 568	Goat	Mouse	polyclonal	Invitrogen	Frederic, MA	A-11004	1:1000
avidin conjugated-HRP				Sigma-Millipore	St.Louis, MO	E2886	1:10000
